# Exposure to Formaldehyde Perturbs the Mouse Gut Microbiome

**DOI:** 10.3390/genes9040192

**Published:** 2018-04-03

**Authors:** Junhui Guo, Yun Zhao, Xingpeng Jiang, Rui Li, Hao Xie, Leixin Ge, Bo Xie, Xu Yang, Luoping Zhang

**Affiliations:** 1 Hubei Key Laboratory of Genetic Regulation and Integrative Biology, School of Life Sciences, Central China Normal University, Wuhan 430079, China; guojunhui@whut.edu.cn (J.G.); zhaoyun@mails.ccnu.edu.cn (Y.Z.); ruili@mail.ccnu.edu.cn (R.L.); glx31415926@163.com (L.G.); xiebo@mail.ccnu.edu.cn (B.X.); 2 Division of Environmental Health Sciences, School of Public Health, University of California, Berkeley, CA 94720, USA; 3 School of Chemistry, Chemical Engineering and Life Science, Wuhan University of Technology, Wuhan 430070, China; h.xie@whut.edu.cn; 4 School of Computer, Central China Normal University, Wuhan 430079, China; xpjiang@mail.ccnu.edu.cn

**Keywords:** formaldehyde (FA), gut microbiome, 16s rRNA gene, high-throughput, murine model

## Abstract

Exposure to Formaldehyde (FA) results in many pathophysiological symptoms, however the underlying mechanisms are not well understood. Given the complicated modulatory role of intestinal microbiota on human health, we hypothesized that interactions between FA and the gut microbiome may account for FA’s toxicity. Balb/c mice were allocated randomly to three groups: a control group, a methanol group (0.1 and 0.3 ng/mL MeOH subgroups), and an FA group (1 and 3 ng/mL FA subgroups). Groups of either three or six mice were used for the control or experiment. We applied high-throughput sequencing of 16S ribosomal RNA (rRNA) gene approaches and investigated possible alterations in the composition of mouse gut microbiota induced by FA. Changes in bacterial genera induced by FA exposure were identified. By analyzing KEGG metabolic pathways predicted by PICRUSt software, we also explored the potential metabolic changes, such as alpha-Linolenic acid metabolism and pathways in cancer, associated with FA exposure in mice. To the best of our knowledge, this preliminary study is the first to identify changes in the mouse gut microbiome after FA exposure, and to analyze the relevant potential metabolisms. The limitation of this study: this study is relatively small and needs to be further confirmed through a larger study.

## 1. Introduction

The diverse populations of bacteria harbored in the human gastrointestinal tract are collectively referred to as the gut microbiome [1]. At birth the human gastrointestinal tract is sterile, but a microbial ecosystem is rapidly established in the human gut [2], eventually hosting up to >1000 bacteria species, which account for 90% of the cells of the human body [3,4,5]. Gut microbiota genome encoding 3.3 million genes are more than 140 times greater than the human genome [6]. It was previously believed that there was not much interaction between the host and the bacteria inhabiting the intestine [7]. However, mounting recent evidence shows that the gut microbiome plays an important role in energy production, food digestion, immune system development and other metabolic processes in the host [8]. The dysbiosis of gut microbiota may contribute to digestive system dysfunction and various diseases, including obesity, inflammatory bowel diseases, diabetes, allergies, arthritic diseases and cardiovascular diseases [9,10,11,12,13]. As a matter of fact, the gut microbiome has the ability to regulate a variety of physiological and metabolic processes in the human body in a profound way. For example, some carbohydrates that humans cannot digest can be biotransformed by the gut microbiome via fermentation to produce short chain fatty acids (SCFAs) which form part of the energy substrates for the colonic epithelium and other peripheral tissues [14].

The composition of the gut microbiota remains stable in the absence of any significant interference after evolving for several years after birth. However, some external factors such as antibiotics and toxic environmental chemicals can perturb the gut microbial community and induce dysbiosis [15,16,17,18,19]. For instance, oral exposure to ochratoxin A (OTA) leads to a diminished diversity of gut microbiota and a relatively high abundance of *Lactobacillus* [16], arsenic induces structural and compositional colonic microbiome change and promotes host nitrogen and amino acid metabolism [15], manganese induces sex-specific gut microbiome perturbations in mice [17], and subchronic oral exposure to benzo[a]pyrene (BaP), the most toxic polycyclic aromatic hydrocarbon (PAH), induces moderate inflammation primarily in the ileal mucosa and alters the relative abundance of both fecal and mucosa-associated microbiota [1]. Disorder in the gut microbiome community induced by exposure to environmental chemicals may be one of the most important mechanisms by which those toxicants could adversely impact human health. Up until now, the environmental pollutants that have been reported to disturb the gut microbiota include: 2,3,7,8-tetrachlorodibenzofuran (TCDF) [18], BaP [1], and diazinon (an organophosphate insecticide) [19] in addition to OTA [16] and arsenic [15]. However, there is no substantial evidence of, nor studies into, any association between the gut microbiome and formaldehyde exposure, to the best of our knowledge.

Formaldehyde (FA), a toxic environmental chemical, is ubiquitously used in many industrial and consumer products. In recent years, the use of FA in China has surged dramatically, leading to severe environmental pollution. The average level of urban indoor air formaldehyde has reached 0.238 mg/m^3^, exceeding the national standard of 0.1 mg/m^3^ [20]. Many toxic responses, including immunotoxicity [21], neurotoxicity [22], genotoxicity [21], hepatic [23] and renal [24] toxicity, have been associated with FA exposure. In 2004, The International Agency for Research on Cancer (IARC) classified FA as a human carcinogen that could cause nasopharyngeal cancer [25], and later in 2010, IARC stated that FA could also cause leukemia, particularly the myeloid type [26]. In view of its widespread use, various toxicity, high volatility, and poor regulation, FA poses a significant danger to human health not only in China and in the USA, but globally [27,28]. Therefore, it is urgent for the public to be made aware of the serious health effects of FA, and it is important for researchers to understand the mechanisms of action of FA-induced toxicity.

A host of studies have been undertaken to elucidate the mechanisms underlying FA-induced cancers and other diseases, such as oxidative stress, DNA damage, chromosomal aberrations and apoptosis [24,29,30]. Given the complex modulatory role of intestinal microbiota on human physiology and the evidence of gut microbiome disturbance reportedly associated with exposures to a few other environmental chemicals, here we hypothesized that exposure to FA could also disturb the gut microbiota landscape, which may play an important role in human health outcomes (e.g., disease) induced by the chemical.

To test the hypothesis, we applied high-throughput sequencing of 16S ribosomal RNA (rRNA) genes to an experimental mouse model to investigate whether FA exposure can potentially alter the composition of the gut microbiota, and which specific types or classes or families of the microbiome are observed in those FA-caused landscape changes. Applying bioinformatic analyses with KEGG (Kyoto Encyclopedia of Genes and Genomes, http://www.genome.jp/kegg/) and PICRUSt (http://picrust.github.io/picrust/) software programs [31], we further explored and predicted relevant metabolic pathways involved. Our preliminary data revealed the microbiome changes in the gut of FA-exposed mice and the main types and levels of (bacterial family and genus levels) bacteria associated with the exposure. However, these findings need to be further confirmed in future studies.

## 2. Materials and Methods

### 2.1. Experimental Animals and Design

Balb/c mice (weighing about 20 g, six weeks of age) from the same parents were purchased from Hubei Research Center of Laboratory Animals (Wuhan, China). Before and throughout the experimental period, mice were provided with a standard pelleted rodent diet, filtered water *ad libitum* and were housed in a pathogen-free animal room (24–26 °C) with a 12 h light-dark cycle, and 55–75% humidity. To avoid coprophagia and other cross-contamination, each single mouse was housed in a separate cage. All experiments were approved by the Institutional Animal Care and Use Committee of Central China Normal University on 24 January 2016 (Ethics Ratification ID: CCNU-IACUC-2016-003). Balb/c mice were allocated randomly to three groups: unexposed control group (saline solution only, six mice), MeOH group (including two subgroups: 0.1 and 0.3 ng/mL MeOH subgroups, three mice each subgroup), and FA group (including two subgroups: 1 and 3 ng/mL FA subgroups, six mice each subgroup). Since this study is in the preliminary research stage and animal research ethics were also a cause of concern, groups of either three or six mice were used for the control or experimental groups [16]. FA was purchased as 10% neutral buffered formalin solution (Sigma-Aldrich Co., Shanghai, China). Since MeOH is usually added in the formalin solution to prevent polymerization of FA, we included the MeOH as an additional control group. All mice had been intragastrically administered either saline solution, MeOH, or FA at different levels for 24 consecutive days. During this exposure period, the mice were weighed daily, and feces from each mouse were carefully collected on day 24 and stored at −80 °C prior to DNA extraction.

### 2.2. 16S Ribosomal RNA Gene Sequencing

Total DNA from the collected fecal pellets was extracted using the E.Z.N.A. DNA kit (Omega Bio-Tek, Norcross, GA, USA) according to the manufacturer’s instructions. All DNA samples were amplified by PCR using 5-TCGTCGGCAGCGTCAGATGTGTATAAGAGACAGCCTACGGGNGGCWGCAG and 5-GTCTCGTGGGCTCGGAGATGTGTATAAGAGACAGGACTACHVGGGTATCTAATCC to target the V3–V4 regions of the 16S rRNA gene of the gut microbiota. The DNA library was constructed via barcoding the resultant DNA according to Illumina standard protocol (see metadata). Sequencing was performed using an Illumina MiSeq platform (Illumina, Santiago, California, USA) to generate 2X300bp pair-ended reads in the School of Life Science facilities of Central China Normal University.

### 2.3. High-Throughput 16S rRNA Gene Sequencing Data Analysis

FLASH [32] was used to merge the paired-end reads. Quantitative Insights into Microbial Ecology software (QIIME, 1.9.1, [33]) was used for quality-filtering and demultiplexing the sequences at the Phred quality score ≥ 20. Operational taxonomic units (OTUs) were picked up with the open-reference approach using UCLUST [34] at a threshold of 97% similarity with the Greengenes (13.8) reference [35]. ChimeraSlayer [36] was used for the detection and removal of chimeric OTUs. We kept only those OTUs with ≥2 reads in at least three repeats of one treatment to improve the consistency of the sequenced data. We obtained a total of 1197 OTUs from 699,775 reads after the pre-processing. For all representative 16S rRNA gene sequences, the classification was processed at different levels using the UCLUST method.

### 2.4. Statistical and Bioinformatic Analysis

For Alpha diversity and Beta diversity assays, the OTUs were rarefied to 29,276 reads per sample. Alpha diversities (chao1 richness and phylogenetic metrics), Beta diversities (Bray-Curtis matrix) and Principal Coordinate Analyses (PCoA) were analyzed using the QIIME and Vegan package in R program [37]. Furthermore, non-negative matrix factorization was employed to identify positively interacting OTU clusters on the 16S rRNA gene data [38]. Heatmaps were constructed by using the software HemI [39] to illustrate the gut bacterial taxa changes at phylum, family and genus levels induced by FA or MeOH treatment.

Metagenomic content of the samples was predicted from the 16S rRNA profiles, and subsequently the KEGG pathways were categorized using the phylogenetic information by reconstructing the whole microbial community (PICRUSt, default parameters) [40]. The metagenomic data was further analyzed by using the STAMP v2.1.3 (Welch’s *t*-test, Two-sided) to reveal differences between the groups [41].

## 3. Results

After FA treatment (at 1 and 3 ng/mL), the mice exhibited some signs of toxicity, such as weight loss and fatigue (data not shown).

### 3.1. Richness and Diversity of the Mouse Gut Microbiome

From the high-throughput sequencing of 16S rRNA gene, the mouse feces microbiome composition was profiled. Table S1 (see Supplementary Material) shows the summary of sequence data. Figure S1 (see Supplementary Material) shows the relationship between the observed OTU numbers and the fecal samples. The number of observed OTUs ranged from 795 (3.0 ng/mL FA) to 892 (1.0 ng/mL FA) (Table 1). The alteration indicated by the global ANOVA test was produced by the MeOH and there were no differences between each FA group and its corresponding MeOH group (Table 1, Figure S2, Supporting Information).

### 3.2. Mouse Gut Microbiota Composition Profiles

**Figure 1:**
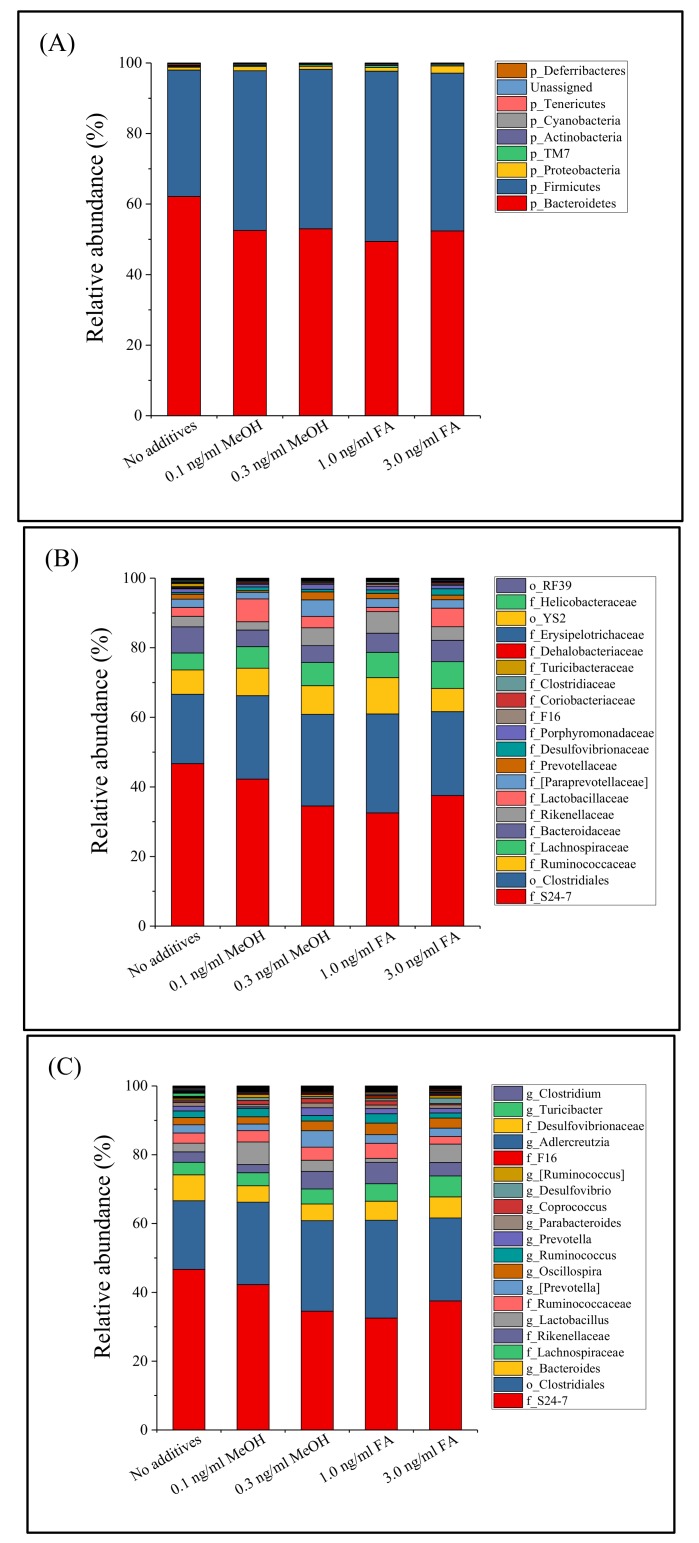
Fecal microbiota composition profiles at the phylum level (**A**), family level ((**B**), top 20 families with the most relative abundance were labeled) and genus level ((**C**), top 20 genera with the most relative abundance were labeled) in mice treated with different doses of MeOH or FA.

Distribution and alteration of the microbe taxa in the different groups of mice (untreated, MeOH or FA treated) were further analyzed. Regardless of the fecal sample considered, *Bacteroidetes* (54.9%, shown in red) and Firmicutes (42.7%, blue) dominated the microbe taxa at the phylum level (Figure 1), followed by Proteobacteria (1.3%), *Actinobacteria *(0.3%), TM7 (0.3%), *Cyanobacteria* (0.1%), *Deferribacteres* (0.1%), and *Tenericutes* (0.1%). This result is similar to that of Dimova et al. [42]. During this study, 55 bacterial genera were observed among the 36 bacterial families detected. A total of 35 bacterial families and 52 genera were common to all of the treated and untreated mice (Figure 1). At the genus level, the fecal microbial community was dominated by unidentified genera from the S24-7 family (38.8%) and the *Clostridiales* order (24.5%). The relative abundance of a bacterial genus (*Turicibacter*) was greatly reduced in the MeOH or FA treated samples.

### 3.3. Microbial Taxa Changes Associated with FA Treatment

**Figure 2:**
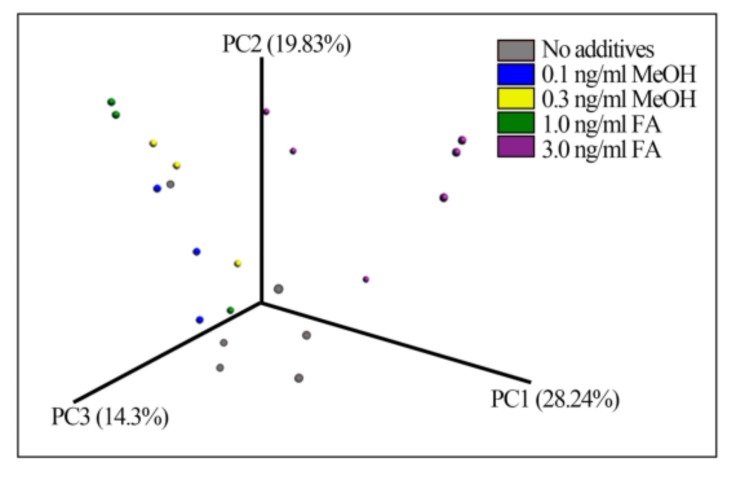
Fecal bacterial patterns of FA-treated mice differentiated by principal coordinate analysis (PCoA) on a Bray-Curtis distance matrix. The patterns show the bacterial taxa community after treatment with different doses of FA or MeOH for 24 days.

Beta-diversity measured by Bray–Curtis distance metrics showed significant differences between the treatments (ANOSIM test *p*-value = 0.02, permutations 999) (Figure 2), suggesting that treatment with FA or MeOH impacted the mouse gut microbial community. The bacterial taxa composition of samples from the different FA-treated mice fall into different groups.

**Figure 3:**
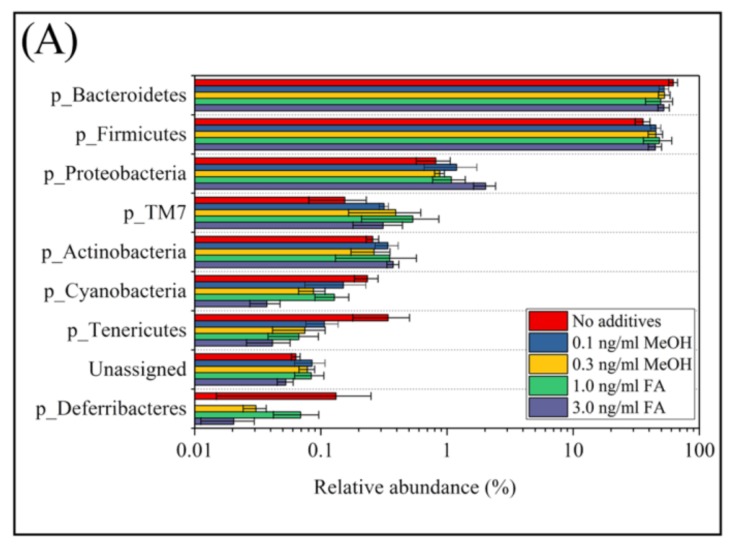

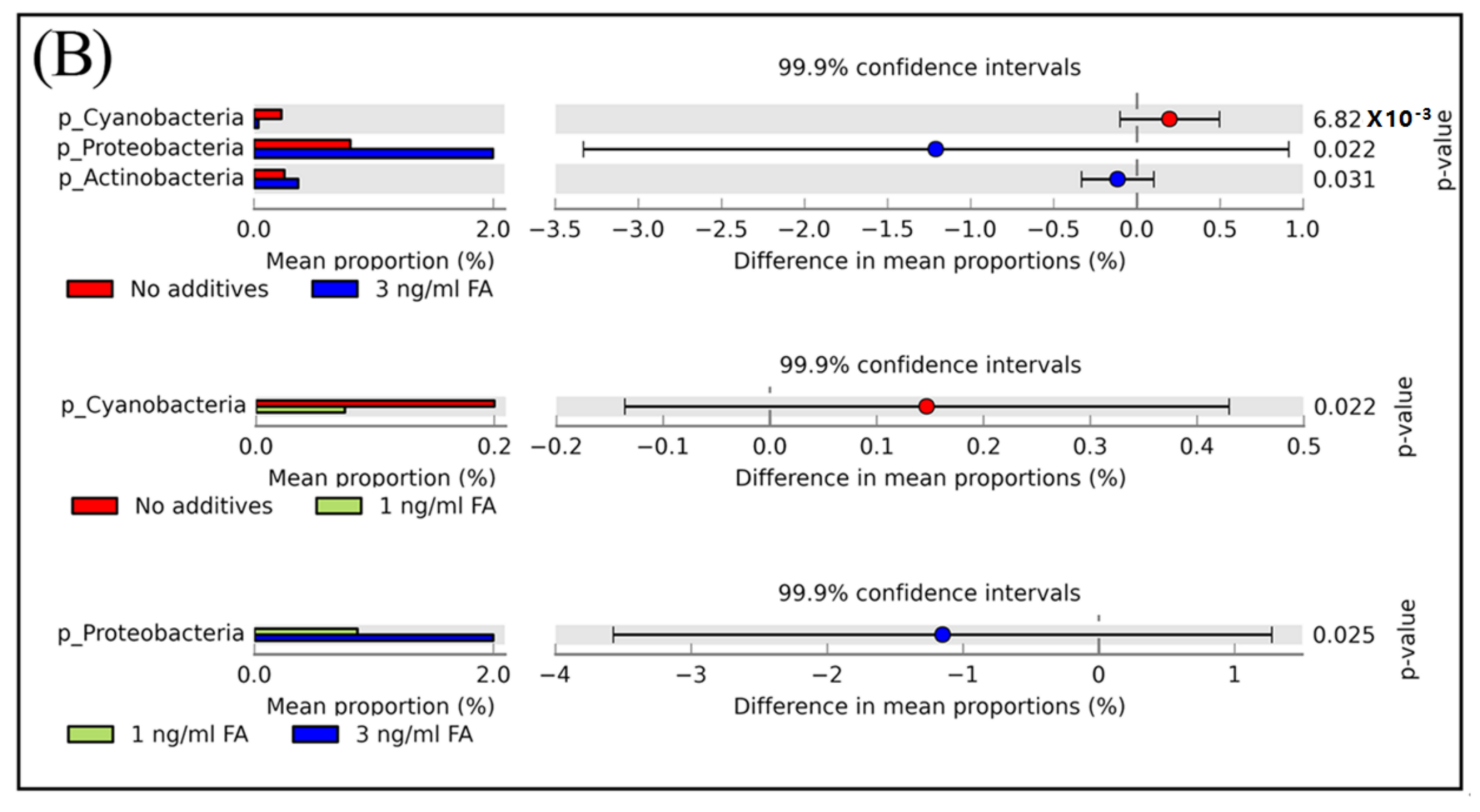
The relative abundance of bacterial community composition at the phylum level (**A**) and different phyla between untreated and FA treated mice detected by STAMP software [41] (**B**). In Panel (**A**) error bars are illustrated as standard deviations. In Panel (**B**) comparisons of the relative abundance of bacterial community composition were made between the 3 ng/mL FA group and the untreated group (**top**), between the 1 ng/mL FA group and the untreated group (**middle**), and between the 1 ng/mL group and the 3 ng/mL group (**bottom**). For each comparison, the mean proportion of phyla (**left**) and difference in mean proportions (**right**) were illustrated.

According to their relative abundance, members of the bacterial community can be categorized as: rare members (<0.1% in relative abundance), common members (between 0.1% and 1%), and abundant members (>1% in relative abundance) [1]. Among the eight phyla observed in mice feces with or without FA or MeOH treatment, the two dominant phyla were Bacteroidetes and Firmicutes. The three next most common phyla were Proteobacteria, TM7, and Actinobacteria, and the three rarest phyla were Cyanobacteria, Tenericutes, and Deferribacteres (Figure 3, Panel A). There were a small number (<0.1% in relative abundance) of unassigned OTUs. Shift of phyla between untreated and FA or MeOH treated mice groups were analyzed by STAMP software [41] (Figure 3B). No significant difference was detected between untreated and MeOH treated mice groups. However, increases in the relative abundance of two phyla (*Proteobacteria* and *Actinobacteria*) and decrease in the relative abundance of phylum *Cyanobacteria* were observed when mice were subjected to treatment of 3.0 ng/mL FA. Since decrease in the relative abundance of *Cyanobacteria* was also observed when mice were subjected to treatment of 1.0 ng/mL FA, it implied that *Cyanobacteria* were affected by the presence of FA in the mouse gut. An increase in the relative abundance of *Proteobacteria* was observed in the mice treated with 3.0 ng/mL FA when compared to the mice treated with 1.0 ng/mL FA.

Shifts of fecal microbial composition were then examined at the genus level. Among the 55 genera detected in this study, 12 dominant genera exhibited average relative abundance over 1% (Figure S3, Panel A). Fifteen genera were detected as common genera with average relative abundance between 0.1% and 1% (Figure S3, Panel B). The other 28 genera were considered as rare, with average relative abundances less than 0.1% and 1% (Figure S3, Panels C and D).

**Figure 4:**
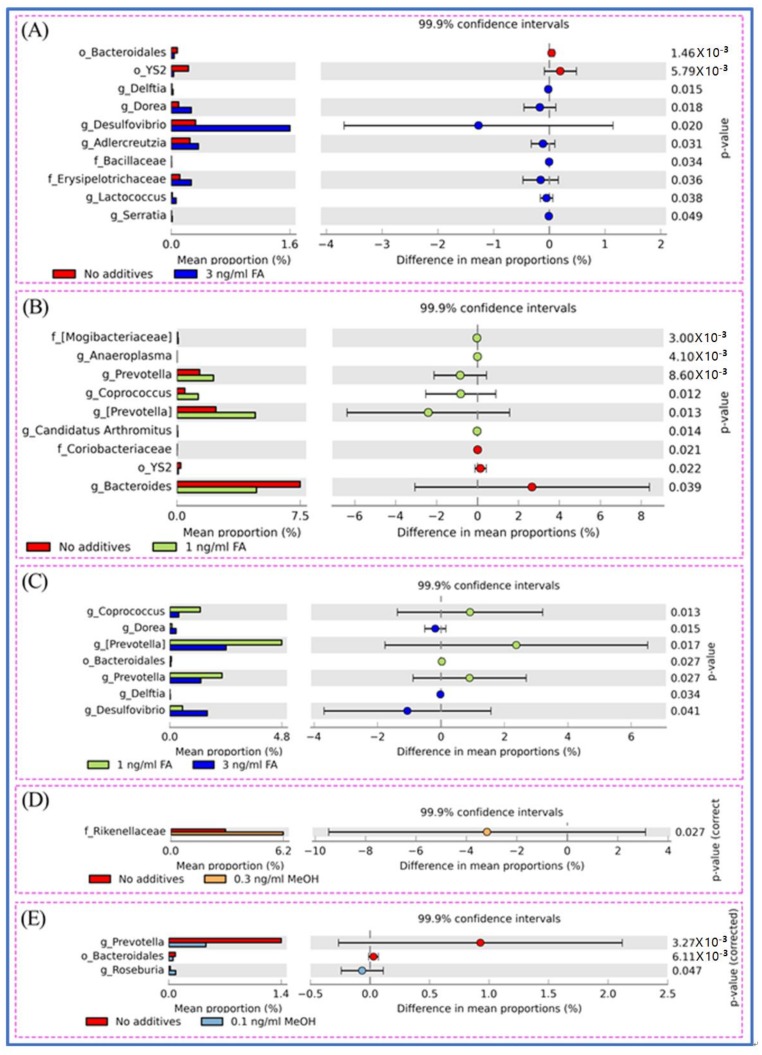
Different genera between untreated and FA treated mice detected by STAMP software [41]. (**A**) Different genera between untreated and the 3.0 ng/mL FA treated mice. (**B**) Different genera between untreated and the 1.0 ng/mL FA treated mice. (**C**) Different genera between the 3.0 and the 1.0 ng/mL FA treated mice. (**D**) Different genera between untreated and the 0.3 ng/mL MeOH treated mice. (**E**) Different genera between untreated and the 0.1 ng/mL MeOH treated mice. For each comparison, the mean proportion of genera (**left**) and difference in mean proportions (**right**) were illustrated.

The shift of genera upon FA or MeOH treatment was analyzed by STAMP software [41]. Figure 4 shows that variation was observed between the FA groups, but not the MeOH groups. The relative abundance of bacterial community composition was compared between the 3 ng/mL FA and untreated groups (Figure 4A), between the 1 ng/mL FA and untreated groups (Figure 4B), and between the 1 ng/mL and 3 ng/mL groups (Figure 4C). After eliminating genera (*Prevotella*, *Bacteroidales*) associated with MeOH treatment, it was observed that there were significant increases in the abundance of thirteen genera and decreases in the abundance of four genera when comparing both FA treated samples (from 1 ng/mL FA to 3 ng/mL FA treated mice) with untreated samples. Among genera with increasing relative abundance, *Prevotella* is one of the dominant genera. Five genera (*Dorea*, *Desulfovibrio*, *Adlercreutzia*, *Anaeroplasma*, *Coprococcus*) and an unidentified member of the *Erysipelotrichaceae* family are common members. Four genera (*Candidatus Arthromitus*, *Delftia*, *Lactococcus*, *Serratia*) and two unidentified genera (from the *Bacilliaceae* family and the *Mogibacteriaceae* family) are rare members. The four genera with decreasing abundance upon FA treatment are *Bacteroides* and three unidentified genera (from the *Bacteroidales* order, the YS2 order, and *Coriobacteriaceae* family). These genera include one dominant member (*Bacteroides*), one common member (from YS2 order), and two rare members (from *Bacteroidales* order and *Coriobacteriaceae* family).

**Figure 5:**
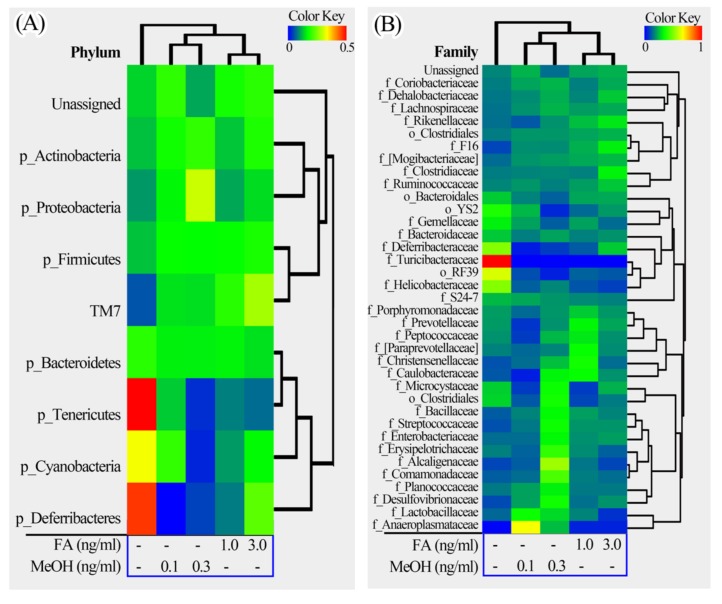

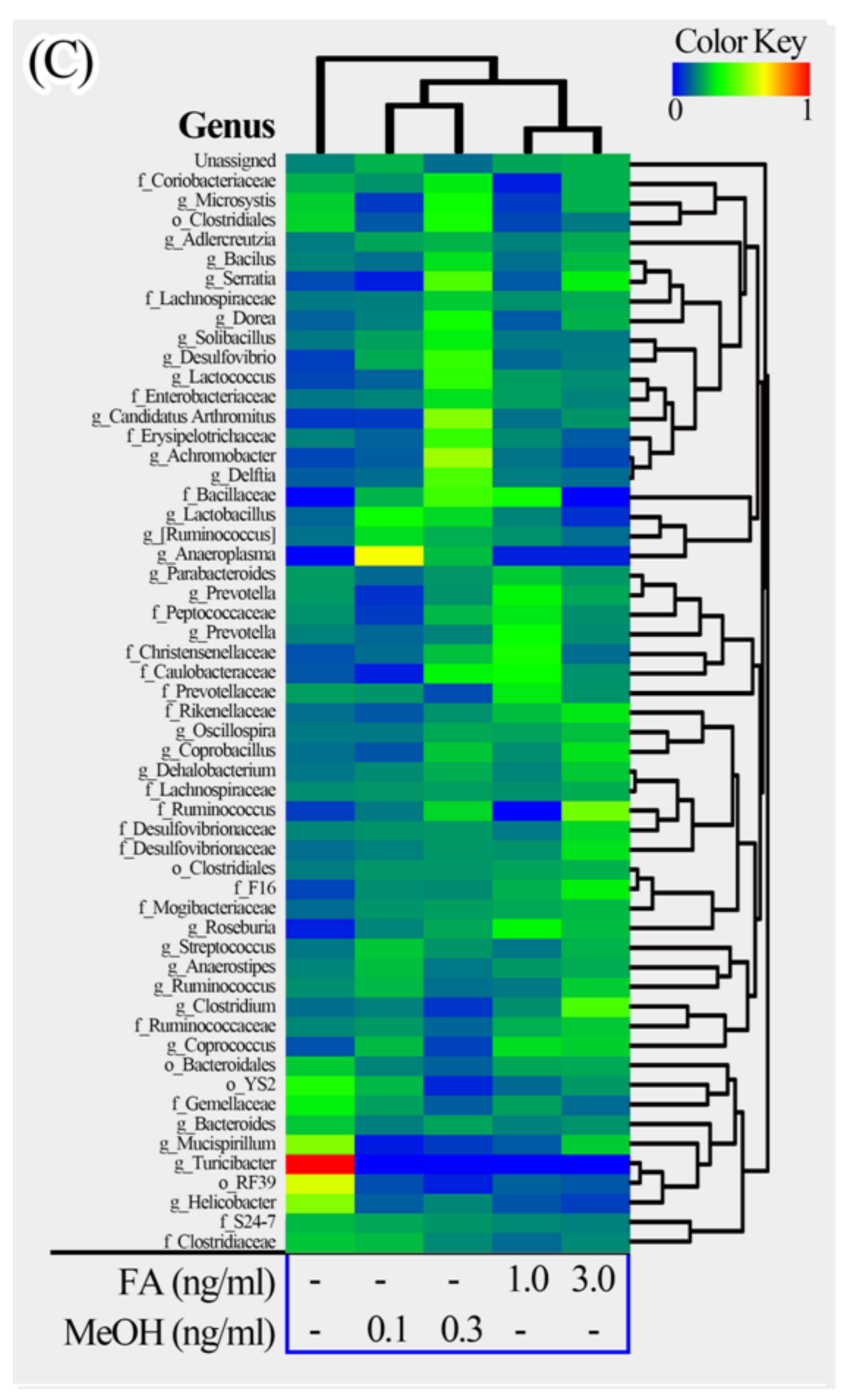
Heatmap illustrations of gut bacterial taxa changes at phylum, family and genus levels. The heatmaps were constructed using the software HemI [39]. (**A**) Phylum; (**B**) Family; (**C**) Genus.

The relative richness of the fecal samples from the FA or MeOH treated mice was further analyzed using heatmap illustrations (Figure 5). The taxa similarity of the fecal samples was investigated by clustering analysis.

The phyla could be divided into two major clusters. One cluster includes *Actinobacteria*, TM7, *Firmicutes*, *Proteobacteria*, and some unassigned bacteria. *Firmicutes* is the dominant phylum in this cluster. The other cluster includes *Bacteroidetes*, *Tenericutes*, *Cyanobacteria*, *Deferribacteres. Bacteroidetes* is the dominant phylum in this cluster. The families could be divided into four major clusters. The genera could be divided into seven major clusters. Based on the clustering analysis, we found that the MeOH or FA treated samples share higher similarity than those from untreated samples at the phylum, family and genus levels. Samples from MeOH groups shared higher similarity than samples from FA groups.

### 3.4. Functional Capacity Profiling Prediction of the Gut Microbiome

**Figure 6:**
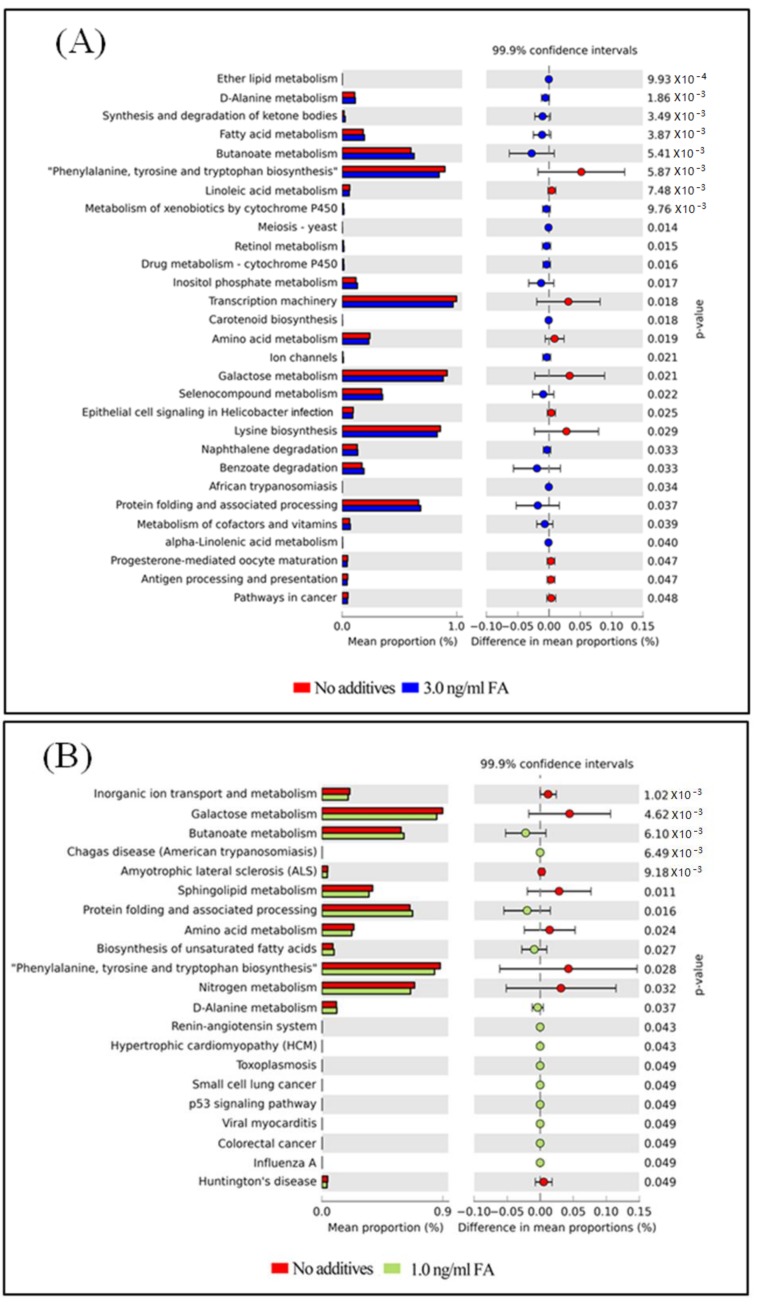

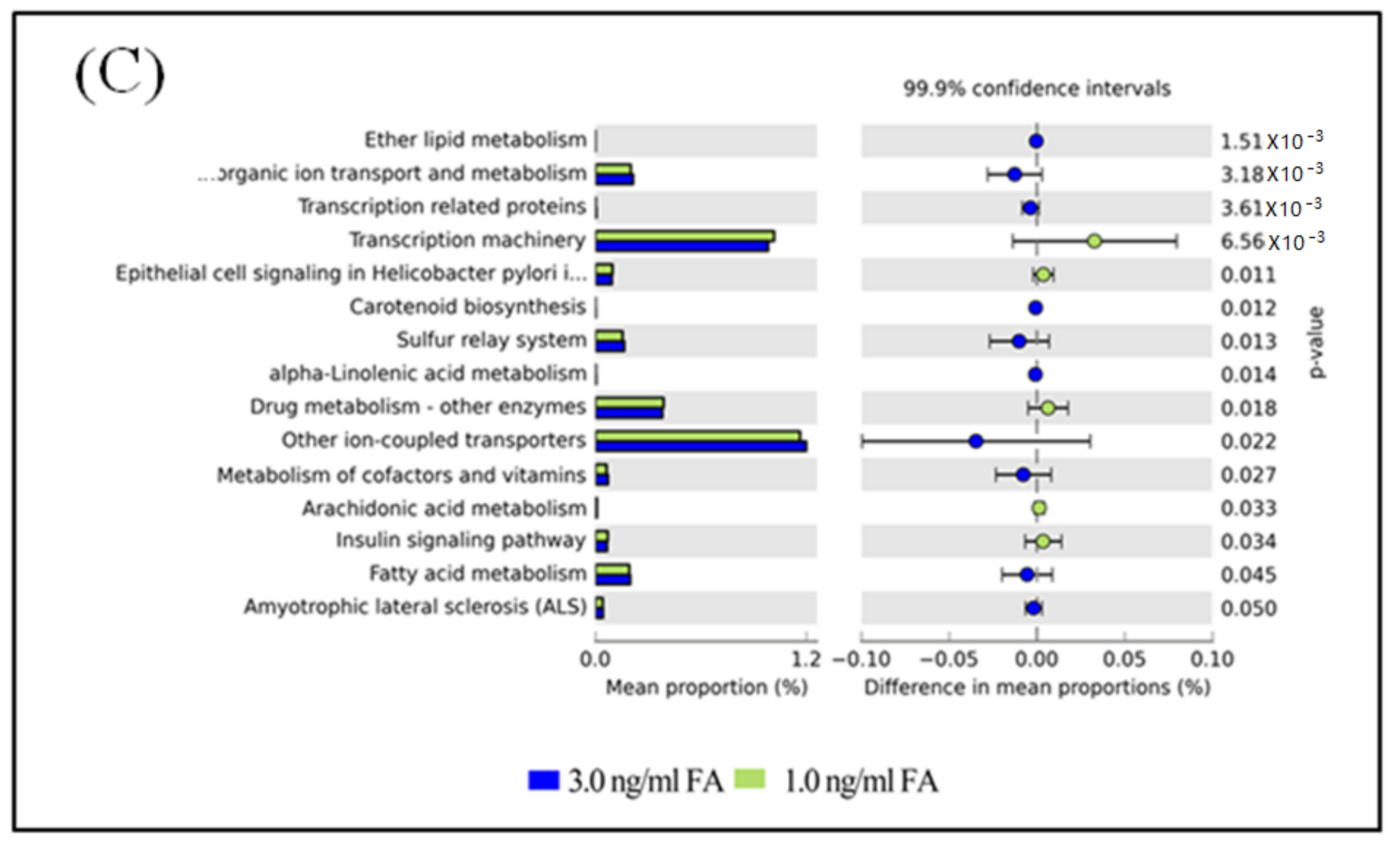
Differential PICRUSt predicted KEGG pathways between untreated and FA treated mice detected by STAMP software [41]. (**A**) Differential KEGG pathways between untreated and the 3.0 ng/mL FA treated mice. (**B**) Differential KEGG pathways between untreated and the 1.0 ng/mL FA treated mice. (**C**) Differential KEGG pathways between the 3.0 and the 1.0 ng/mL FA treated mice. For each comparison, the mean proportion of predicted KEGG pathways (left) and difference in mean proportions (right) were illustrated.

The functional capacity of the gut microbiome after FA or MeOH treatment was evaluated with PICRUSt software [40] based on 16S rRNA profiles. A total of 251 KEGG pathways that may be associated with FA and/or MeOH treatment were detected. Twenty-nine KEGG pathways showed distinct changes between untreated and the 3.0 ng/mL of FA treated mice (Figure 6, Panel A). Twenty-two KEGG pathways exhibited distinct changes between untreated and the 1.0 ng/mL of FA treated mice (Figure 6, Panel B). Sixteen KEGG pathways exhibited distinct changes between the 1.0 ng/mL of FA treated mice and the 3.0 ng/mL of FA treated ones (Figure 6C).

After eliminating pathways that may be associated with MeOH treatment (Figure S4), it was observed that there were increases in the abundance of twenty KEGG pathways and decreases in the abundance of eight KEGG pathways when comparing both FA treated samples (from 1 ng/mL FA to 3 ng/mL FA treated mice) with untreated samples.

Increases in the abundance of eight KEGG pathways and decreases in the abundance of five KEGG pathways were detected when comparing the 3.0 ng/mL of FA treated samples with the 1.0 ng/mL of FA treated samples or with the untreated samples. Although six KEGG pathways were detected when comparing untreated sample with FA treated sample, changes in seven KEGG pathways were observed between 3.0 ng/mL and 1.0 ng/mL FA treated samples. We suspected that these pathways were also associated with FA treatment.

## 4. Discussion

Associations among human diseases, gut microbiota and exposures to environmental pollutants (such as FA) have been previously reported [9,10,11,26,27]. Emerging evidence suggests that environmental factors may have effects on the composition of gut microbiota [1]. In this study, the potential effects of FA exposure at high dosage (3.0 ng/mL) and low dosage (1.0 ng/mL) were investigated on the gut bacterial structure in mice. Control groups included untreated mice and MeOH treated mice. FA is a molecule characterized by toxic properties. Impact of FA was found on the bacterial richness or diversity of stools in mice. This result is similar to what has been observed for other environmental pollutants such as polychlorinated biphenyls (PCBs) or heavy metals [43,44].

Although the physiological and anatomical structures of humans and mice are very similar, and the mechanisms of interactions between microbial communities and hosts are shared among most of these species, there are some key differences between mouse models and humans. For example, although 85% of the bacterial species found in mice do not exist in humans [12], a similar trend was observed in the composition of the intestinal microflora [45].

In this study, the *Firmicutes* and *Bacteroidetes* phyla dominated the fecal bacterial communities, which is consistent with previous studies [46,47]. Although bacterial populations showed wide diversity and taxonomic composition, they were different in mouse groups and treatments, but the general structure of bacterial assembly remained unchanged. The composition of the fecal bacterial community was mainly composed of a few taxa. Exposure to FA seems to cause environmental stress mainly in the bacterial family and genus levels. At the genus level, a significant increase was observed in the abundance of nine genera in the identified gut microbiota of mice treated with FA. These bacteria were substantial contributors to the functioning of the ecosystem [48].

Although there have been many reports of FA inhalation and resultant symptoms, FA ingestion has only rarely been described in the literature. Hawley and Harsch [49] reported early clinical and endoscopic manifestations appeared relatively mild after FA ingestion and the patient only developed signs and symptoms suggestive of gastric outlet obstruction after several weeks. Another report suggested the FA may have irritated the gastrointestinal tract causing smooth muscle and mucosal inflammation [50]. However, FA inhalation can induce pulmonary changes including disordered alveolar structures, inflammatory cell infiltration, edema in alveolar spaces, congestion and hemorrhage [51,52]. The damage caused by FA to organ tissues may be associated with oxidative stress and inflammation [53,54,55,56]. In the present study, some bacteria species responding to FA treatment might be related to inflammation and other pathological changes, implying microbial shifts in the mouse gut might aggravate direct damages by FA.

We found that there was an increased abundance of thirteen genera. Among these genera, *Prevotella* is one of the dominant genera in the mouse gut. Changes in *Prevotella* are related to the development of osteomyelitis. [57] *Erysipelotrichaceae* is observed to be associated with Major Depressive Disorder [58]. *Desulfovibrio* is a genus of sulfate-reducing bacteria that stimulate gut immune responses and contribute to inflammation in experimental colitis [59]. *Dorea* was reported with a remarkable change of increases in the relative abundance in stool microbiomes during a course of exclusive enteral nutrition in paediatric Crohn’s Disease [60]. *Adlercreutzia* is capable of metabolizing isoflavones to equol [61]. *Anaeroplasma* and *Coprococcus* are two genera occasionally reported in gut mirobiota [62]. 

*Candidatus Arthromitus* is a group of morphologically distinct bacteria found almost exclusively in terrestrial arthropods as filamentous bacterial forms associated often with the gut wall [63]. *Delftia* is a genus of *Comamonas* bacteria. There have been reports of isolation of the organism from serious infections like central venous catheter associated bacteremia, corneal ulcers and otitis media [64]. *Lactococcus* is a genus of lactic acid bacteria that are able to produce lactic acid, the main, or only, product of glucose fermentation. *Serratia* belongs to the *Enterobacteriaceae* family. The bacterium is an opportunistic human pathogen and responsible for about 2% of nosocomial infections of the bloodstream, lower respiratory tract, urinary tract, surgical wounds, and skin and soft tissues in adult patients [65].

Among bacteria with decreased relative abundance upon FA treatment, the *Bacteroides* are members of the human gut microbiota that confer myriad benefits on their hosts. These bacteria are involved in polysaccharide metabolism and utilization [66]. The *Coriobacteriia* are associated with human oral infections as well as severe blood bacteraemia and ulcerative colitis [67,68,69].

The metabolic properties of gut microbiota were inferred from the PICRUSt predicted KEGG pathways. Increases and decreases have been observed in the abundance of many KEGG pathways, which may present a general response of gut microbiomes to environmental stress. Alpha-Linolenic acid (ALA) is an n-3 fatty acid. It is one of two essential fatty acids (the other being linoleic acid), so called because they are necessary for health but cannot be produced within the human body. It is interesting to understand the role of “alpha-Linolenic acid metabolism” in resistance to changes caused by FA exposure. Similarly, some cofactors and vitamins might be affected by treating mice with FA. Therefore, the metabolism of cofactors and vitamins might also be affected. Inositol phosphates are a group of mono- to poly-phosphorylated inositols. They play crucial roles in diverse cellular functions, such as cell growth, apoptosis, cell migration, endocytosis, and cell differentiation. Increases of the two KEGG pathways “Inositol phosphate metabolism” and “Transcription related proteins”, suggest that metabolisms relating to specific proteins might be improved. Naphthalene and Seleno compounds are both toxic. The increase in “Naphthalene degradation” and “Seleno compound metabolism” pathways implies that a detoxification pathway might be activated. The increase of “Ion channels” and “Other ion-coupled transporters” pathways implies there might be increased communication between cells and environments after FA treatment.

A decrease in several KEGG pathways was also observed. A decrease in the pathway “Epithelial cell signaling in Helicobacter pylori infection” might be related to a decrease of richness of *Helicobacter*. A decrease in “Antigen processing and presentation”, “Linoleic acid metabolism”, and “Lysine biosynthesis” pathways might be related to FA influencing normal metabolisms.

Disorders of the KEGG pathways colorectal cancer, “p53 signaling pathway”, “pathways in cancer”, “small cell lung cancer”, and “sulfur relay system” might be related to the carcinogenic mechanism of FA. Disorders of the KEGG pathways “hypertrophic cardiomyopathy (HCM)”, “Iifluenza A”, “viral myocarditis”, “african trypanosomiasis”, “toxoplasmosis”, “chagas disease (American trypanosomiasis)”, “insulin signaling pathway”, and “renin-angiotensin system” might explain the serious adverse health effects caused by FA exposure. Disorders of the KEGG metabolism pathways “biosynthesis of unsaturated fatty acids”, “D-alanine metabolism”, “ether lipid metabolism”, “protein folding and associated processing”, “steroid biosynthesis”, “arachidonic acid metabolism”, and “drug metabolism—other enzymes” indicated that FA treatment might affect a variety of metabolisms and result in complicated damage to the host. More investigations are needed to address the disorders of “progesterone-mediated oocyte maturation”, “transcription machinery” and “photosynthesis—antenna proteins” pathways.

The limitation of this study: the low number of animals makes statistical analysis less robust, except in the case of phylum level variations. We need further confirmation from a larger study.

## 5. Conclusions

This study explored FA-induced alterations to the mouse gut microbiome using the high-throughput sequencing of 16S rRNA gene and metagenomics approaches. Alterations were observed in bacterial richness and diversity of the fecal microbiota after exposure. However, the relative richness was significantly affected in the abundance of several bacterial genera and families as well as several PICRUSt predicted KEGG pathways. The affected genera included abundant members as well as rare members in the bacterial community. A number of KEGG pathways including detoxification and signaling pathways might be affected by FA exposure.

## Figures and Tables

**Table 1:** Richness (Operational taxonomic units, OTU number, Chao1) and diversity (Phylogenetic Diversity, PD) values for fecal-associated bacteria in the formaldehyde (FA) or MeOH treated mouse groups.

	Saline Control	0.1 ng/mL MeOH	0.3 ng/mL MeOH	1.0 ng/mL FA	3.0 ng/mL FA	*p*-Value *
OTUs	833.8 ± 33.7	892.7 ± 15.7	887.3 ± 26.6	881.6 ± 27.9	795.5 ± 69.5	0.046
Chao1	929.2 ± 54.0	1011.8 ± 44.6	993.8 ± 35.6	1018.7 ± 21.0	915.3 ± 51.7	0.023
PD	35.8 ± 1.4	38.0 ± 0.5	37.9 ± 0.7	37.8 ± 1.0	35.4 ± 1.5	0.029

* Calculated by ANOVA test. The values are given as mean ± error. The OTUs were defined to have 97% sequence identity. Calculations were made based on rarefied OTU tables at 29,276 sequences from fecal samples.

## Supplementary Materials

The following are available online at http://www.mdpi.com/2073-4425/9/4/192/s1, Table S1: Summary of sequence data; Figure S1: The rarefaction curves for the FA or MeOH treated samples; Figure S2: The richness (Chao1 index) and phylogenetic diversity analysis of the FA or MeOH treated samples; Figure S3: The relative abundance of bacterial community composition at the genus level; Figure S4: Differential PICRUSt predicted KEGG pathways between untreated and MeOH treated mice detected by STAMP software.
