# Supplementary material for: Exposure to Formaldehyde Perturbs the Mouse Gut Microbiome
Source: Genes (Basel). 2018 Apr 3;9(4):192. doi: 10.3390/genes9040192 (PMC5924534; doi:10.3390/genes9040192)
Supplement: Supplementary file 1 [file genes-09-00192-s001.pdf]

genes

Supplementary Materials

Exposure to Formaldehyde Perturbs the Mouse Gut Microbiome

Junhui Guo [1,2§,4], Yun Zhao [1], Xingpeng Jiang [3,2§], Rui Li [1,2§], Hao Xie [4], Leixin Ge [1], Bo Xie [1,2§], Xu Yang [1,*] and Luoping Zhang [2,*]

[1] Hubei Key Laboratory of Genetic Regulation and Integrative Biology, School of Life Sciences, Central China Normal University, Wuhan 430079, China
[2] Division of Environmental Health Sciences, School of Public Health, University of California, Berkeley, California, USA
[3] School of Computer, Central China Normal University, Wuhan 430079, China
[4] School of Chemistry, Chemical Engineering and Life Science, Wuhan University of Technology, Wuhan 430070, China

§ : As a visiting scholar at UC Berkeley.

* Correspondence: Luoping Zhang, Division of Environmental Health Sciences, School of Public Health, University of California, Berkeley, California, USA. E-mail: luoping@berkeley.edu, Tel.: +1 510 334 5959; and Xu Yang, School of Life Sciences, Central China Normal University, Wuhan 430079, China. E-mail: yangxu@mail.ccnu.edu.cn, Tel.: +86 27 6786 6997.

Supplementary Materials:

Figure S 1.

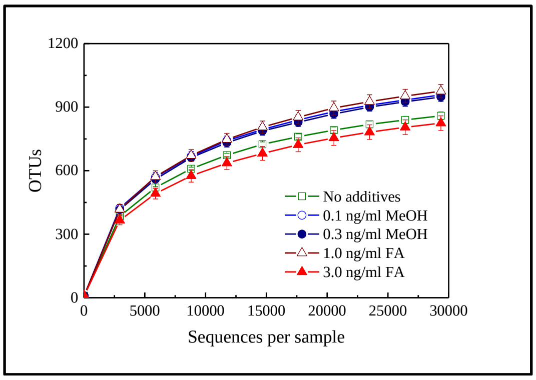

Figure S 1. The rarefaction curves for the FA or MeOH treated samples.

Figure S 2.

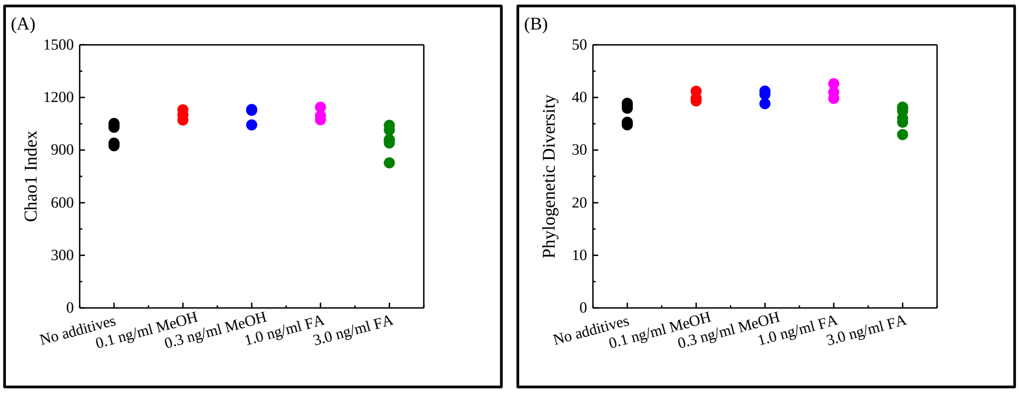

Figure S 2. The richness (Chao1 index) and phylogenetic diversity analysis of the FA or MeOH treated samples.

Figure S 3.

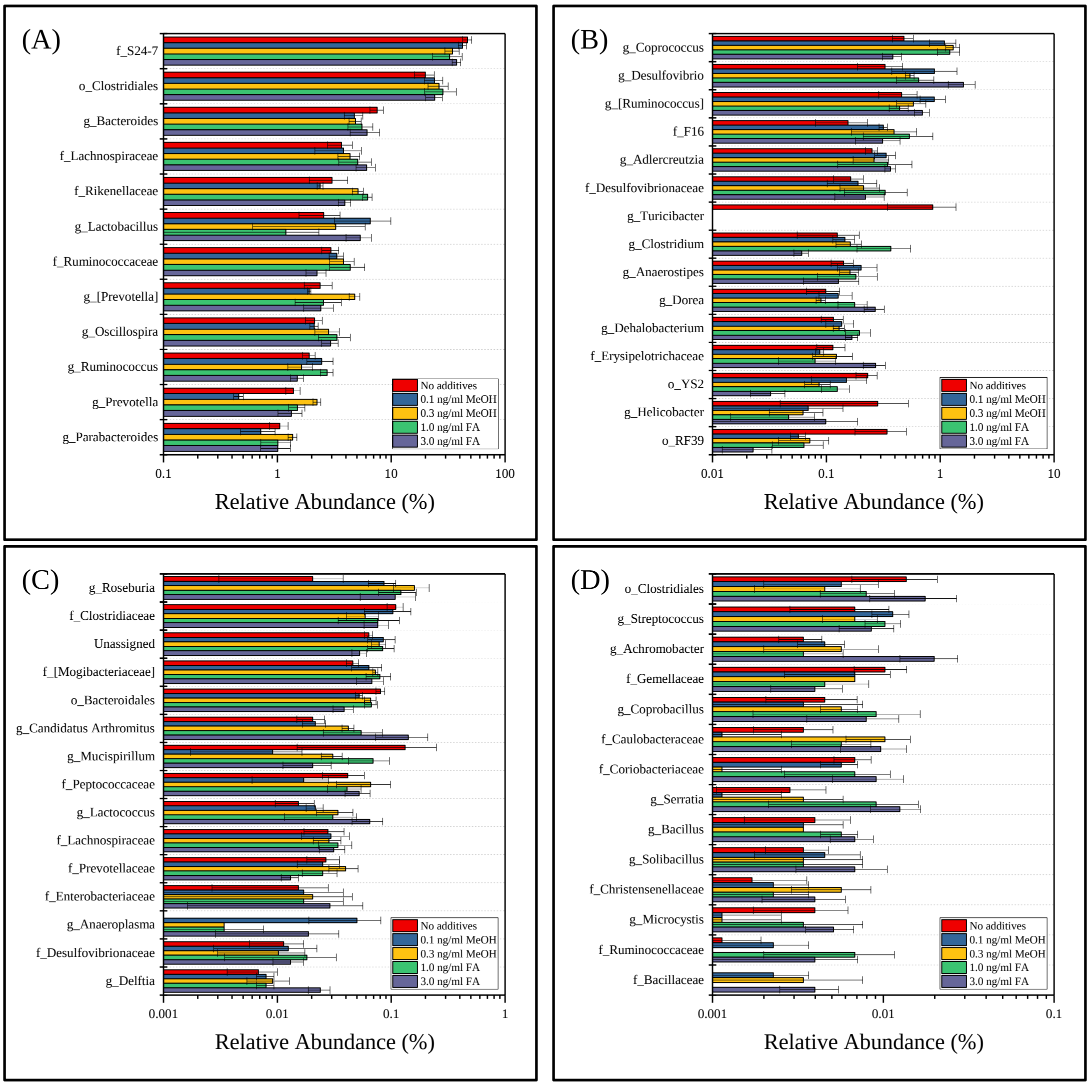

Figure S 3. The relative abundance of bacterial community composition at the genus level. (A), dominant genera; (B), common genera; (C) and (D), rare genera.

Figure S 4.

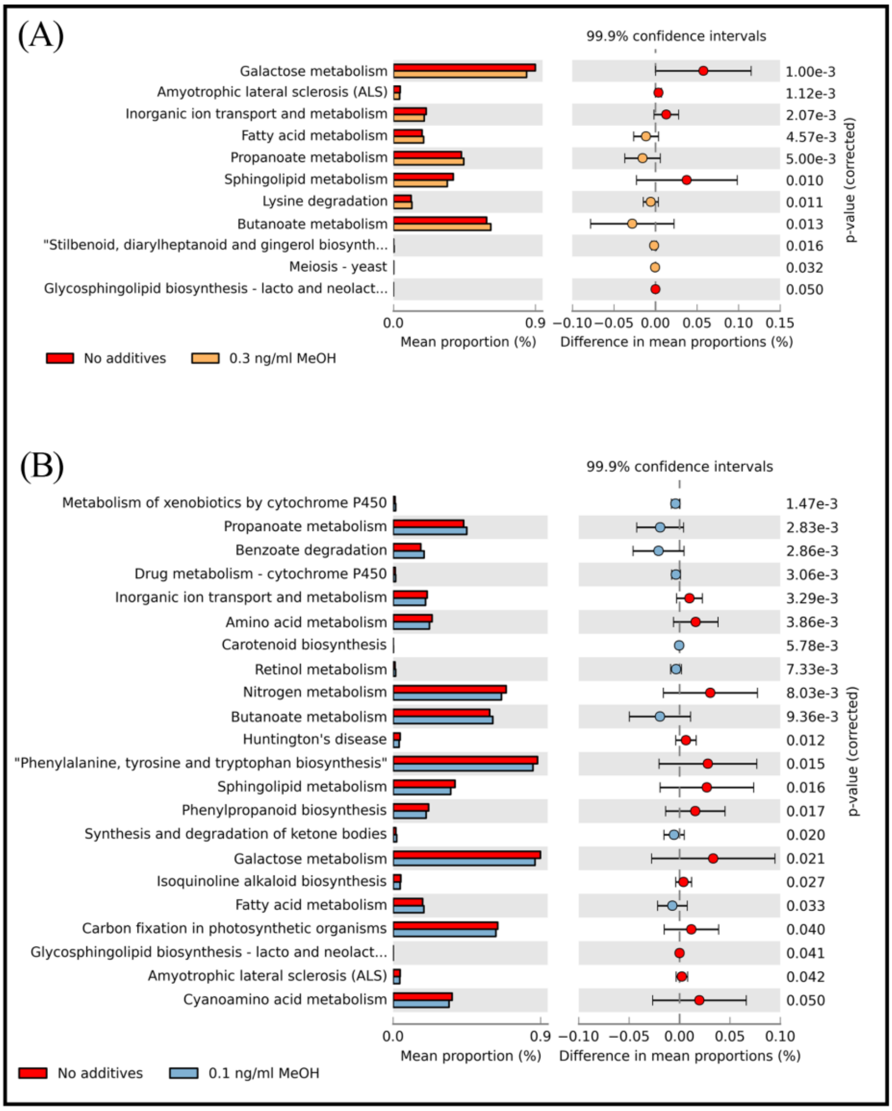

Figure S 4. Differential PICRUSt predicted KEGG pathways between untreated and MeOH treated mice detected by STAMP software [34]. (A) Differential KEGG pathways between untreated and the 0.3 ng/ml MeOH treated mice. (B) Differential KEGG pathways between untreated and the 0.1 ng/ml FA treated mice.

Table S1: Summary of sequence data

Num samples	21
Num observations	1197
Total count	699775
Table density (fraction of non-zero values)	0.723
Counts/sample summary	
Min	29276
Max	38178
Median	33092
Mean	33322.62
Std. dev.	2793.643
Sample Metadata Categories	None provided
Observation Metadata Categories	taxonomy
Counts/sample detail	
p3.24.Y3.3	29276
0.24.Y1.3	29438
p1.24.Y4.1	29650
p10.24.Y4.2	30338
p30.24.Y7.1	30450
p30.24.Y5.3	31165
0.24.Y6.3	31402
0.24.Y6.2	31409
p10.24.Y4.1	31947
0.24.Y6.1	31999
p1.24.Y2.1	33092
p1.24.Y2.3	33961
p30.24.Y7.2	34536
p30.24.Y5.2	34915
p30.24.Y7.3	35349
p3.24.Y3.2	35802
p1.24.Y2.2	35934
p3.24.Y3.1	36462
p30.24.Y5.1	36722
0.24.Y1.1	37750
0.24.Y1.2	38178
